# Early social isolation disrupts adult personality expression in group‐living mites

**DOI:** 10.1111/1365-2656.14169

**Published:** 2024-08-23

**Authors:** Peter Schausberger, Thi Hanh Nguyen

**Affiliations:** ^1^ Department of Behavioral and Cognitive Biology University of Vienna Vienna Austria

**Keywords:** animal personality, group living, predatory mites, sociability, social deprivation, social isolation

## Abstract

1. Animal personalities are characterized by intra‐individual consistency and consistent inter‐individual variability in behaviour across time and contexts. Personalities abound in animals, ranging from sea anemones to insects, arachnids, birds, fish and primates, yet the pathways mediating personality formation and expression remain elusive.
2. Social conditions during the early postnatal period are known determinants of mean behavioural trait expressions later in life, but their relevance in shaping personality trajectories is unknown.
3. Here, we investigated the consequences of early social isolation on adult personality expression in plant‐inhabiting predatory mites *Phytoseiulus persimilis*. These mites are adapted to live in groups. We hypothesized that transient experience of social isolation early in life, that is, deprivation of any social contact during a sensitive window in the post‐hatching phase, has enduring adverse effects on adult personality expression.
4. Newly hatched mites were transiently reared in isolation or in groups and tested as adults for repeatability of various within‐group behaviours, such as movement patterns and mutual interactions including sociability, defined as the propensity to associate and interact benignly with conspecifics, and activity patterns when alone. Groups composed of individuals with the same or different early‐life experiences were repeatedly videotaped and individual behaviours were automatically analysed using AnimalTA.
5. Social experiences early in life had persistent effects on mean behavioural traits as well as adult personality expression, as measured by intraclass correlation coefficients (indicating repeatability). On average, isolation‐reared females moved at higher speeds, meandered less, kept greater distances from others and had fewer immediate neighbours than group‐reared females. Group‐reared females were highly repeatable in inter‐individual distance, moving speed, meandering and area explored, whereas isolation‐reared females were repeatable only in the number of immediate neighbours. Activity, quantified as the proportion of time spent moving within groups, was only repeatable in group‐reared females, whereas activity, quantified as the proportion of time spent moving when alone, was only repeatable in females reared in isolation. Strikingly, also the early‐life experiences of male mates influenced personality expression of mated females, with isolation‐reared males boosting the repeatability of behavioural traits of group‐reared females.
6. Overall, our study provides evidence that a transient phase of social isolation during a critical period early in life has lasting effects that extend into adulthood, impairing adult personality expression. These effects should cascade upward, changing the phenotypic composition and diversity within populations.

## Graphical abstract

This paper is the first to show for any animal that early‐life social isolation disrupts adult personality expression.

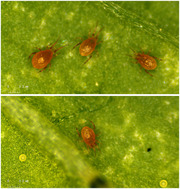

## 1 INTRODUCTION

The social conditions experienced early in life, particularly the presence and identity of interacting genotypes and phenotypes, shape behavioural profiles and can have far‐reaching consequences for later life behaviour and physiology across animals (Langenhof & Komdeur, 2018; Monaghan, 2008; Stamps & Groothuis, 2010; Traniello & Robinson, 2021). A critical social condition is whether or not hatchlings or newborns experience conspecifics during early development. This is particularly relevant for animals that are adapted to living in groups and are frequently interacting with conspecifics. For such animals, social isolation early in life, that is, deprivation of any social contact with conspecifics, can have dramatic adverse impacts on behavioural (Groneberg et al., 2020; Schausberger, Gratzer, & Strodl, 2017), physiological (Cacioppo et al., 2015; Koto et al., 2015; Tanaka et al., 2010), life‐historical (Koto et al., 2023) and/or cognitive (Wang et al., 2022) trajectories, with important evolutionary implications (Bailey & Moore, 2018). For example, social isolation interferes with energy homeostasis and causes oxidative stress that shortens longevity in ants (Koto et al., 2015, 2023), destabilizes brain development in bumblebees (Wang et al., 2022), causes avoidance of social contact in mites (Schausberger, Gratzer, & Strodl, 2017) and zebrafish (Groneberg et al., 2020), and may increase morbidity in humans (Cacioppo et al., 2015). Detrimental long‐lasting effects of social isolation and some of the underlying processes appear to be highly conserved across animal species (e.g. Blanchard et al., 2001; Greek & Rice, 2012) and have enormous relevance to non‐human animal as well as human well‐being and health (House, 2001; House et al., 1988; Snyder‐Mackler et al., 2020; Vora et al., 2022). In humans, objective and perceived social isolation are known to have a critical influence on personality development (e.g. Bowirrat et al., 2023; Cacioppo et al., 2011, 2015; Caspi et al., 2015; Lodi‐Smith et al., 2011; Mund & Neyer, 2019). In non‐human animals, very little is known about the effects of early social isolation on adult personality expression (Gosling, 2001; Liedtke et al., 2015; Réale et al., 2007).

Animals are said to have personalities if they are repeatable in their behaviour, that is, exhibit behaviours that are consistent within individuals while at the same time variable among individuals across time and/or contexts (e.g. Biro & Stamps, 2008; Réale et al., 2007). Research on animal personalities has gained considerable momentum since the early 2000s (Biro & Stamps, 2008; Dall et al., 2004; Gosling, 2001; Réale et al., 2007; Sih et al., 2004). Animal personalities are typically, but not exclusively, evaluated along five major behavioural axes, that is, activity (movement in familiar environments), aggressiveness (hostile behaviour towards conspecifics), boldness (risk‐taking behaviour in familiar environments), exploration (tendency to explore novel environmental features) and sociability (tendency to associate with and exhibit friendly behaviours towards conspecifics) (Réale et al., 2007). Whether and how early social isolation affects the repeatability of behaviour, that is, the formation and expression of animal personality, later in life is largely unknown. This applies particularly to the effects on behaviours displayed in groups, including sociability (Gartland et al., 2022), though this is intuitively the behavioural dimension most likely to be affected. Early‐life conditions are commonly considered those conditions that prevail during the perinatal period, from birth or hatching onwards through the early stages of immature development. The early postnatal period is considered a highly critical, sensitive window in an individual's life (e.g. Andersen, 2003; Bateson, 1979; Carere & Maestripieri, 2013; Fawcett & Frankenhuis, 2015; MacDonald, 1985). The few studies addressing the influence of early social isolation on animal personality traits are difficult to interpret because of social contact among siblings occurring before birth, hatching or emergence, such as within the placenta (Riley et al., 2018 for skinks) or egg sac (Liedtke et al., 2015 for jumping spiders). This can potentially obscure or change subsequent social isolation effects. Information exists for the effects of social isolation just prior to assays (not in early life) on personality expression during assays (Jolles et al., 2016 for exploration in sticklebacks; Skinner et al., 2022 for boldness and sociability in snakes). There is virtually no information available, for any animal, on the effects of early social isolation on the repeatability of sociability and related within‐group behaviours.

**FIGURE 1:**
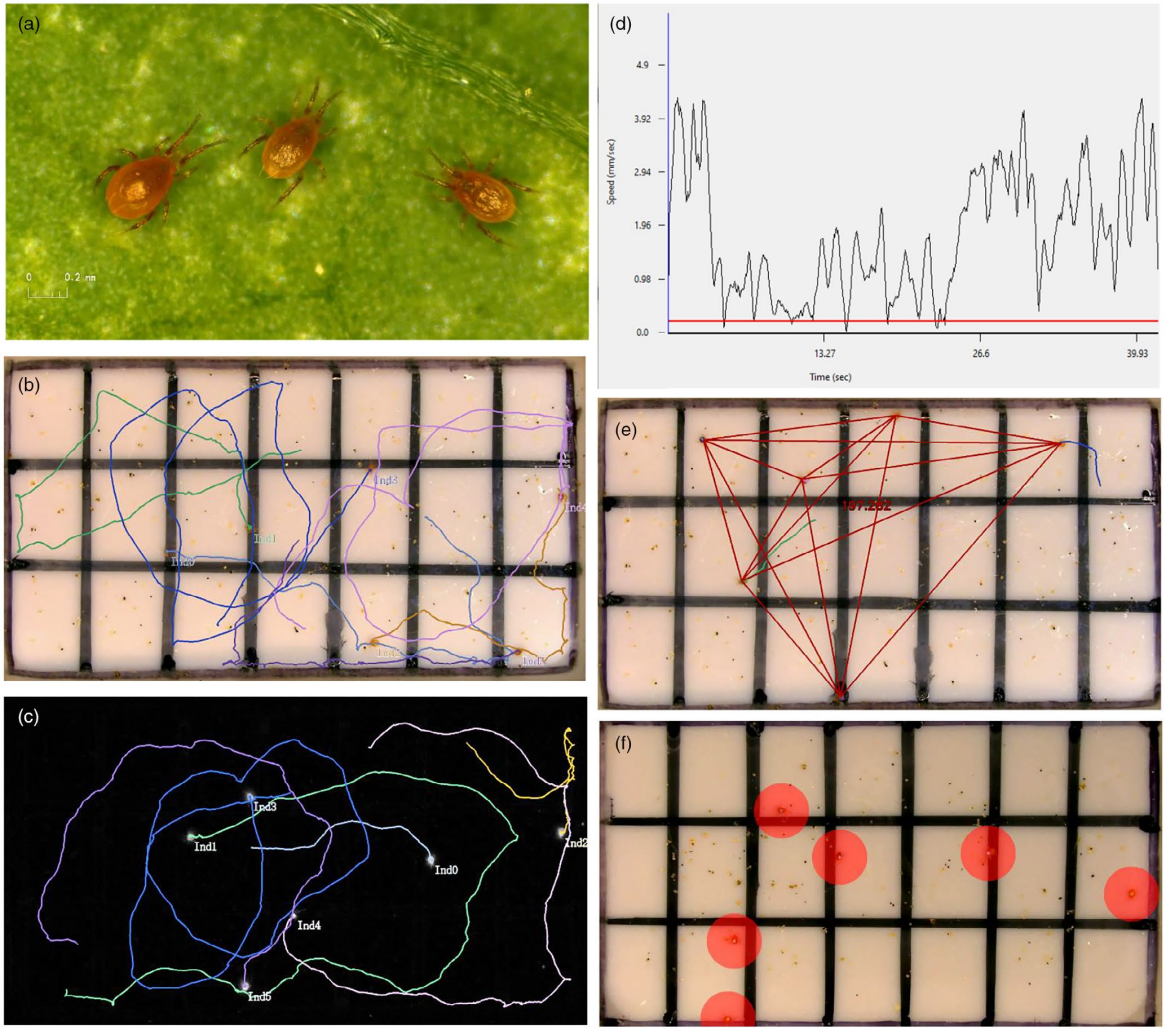
Experimental animals and overview of video‐tracking and analysis features. (a) Adult females of the predatory mite *Phytoseiulus persimilis* on a leaf of common bean, *Phaseolus vulgaris*; (b) acrylic arena (28 × 15 mm) used for videotaping the behaviour of groups of six predatory mite females, with their tracks visualized; (c) arena with background removed and the tracks of the six predatory mites visualized; (d) example of moving speed calculation; (e) example of inter‐individual distance calculation; (f) example of individualized areas used for calculation of the number of immediate neighbours and time spent with ≥1 neighbour [photos (b–f) were extracted from videos analysed by AnimalTA; Chiara & Kim, 2023].

We hypothesized that deprivation of any social contact early in life has lasting effects on adult personality formation. Our prediction was that early‐life social isolation can impair behavioural repeatability when conspecifics are present, that is, disrupts personality expression later in life. Given that critical life‐history events are often tipping points in personality trajectories (e.g. Monestier & Bell, 2020), we pursued the secondary hypothesis that adult female personality expression is also influenced by the behavioural phenotype of the female's mate. We tested our hypotheses in plant‐inhabiting predatory mites *Phytoseiulus persimilis* (Figure 1a). These mites represent a well‐suited system to address our research questions because they are adapted to live in groups and are highly sensitive to environmental stimuli experienced early in life. Egg dispersion depends on prey density; at medium to high prey densities, eggs are typically aggregated in prey patches, while at low prey densities, eggs are more dispersed, resulting in some individuals not encountering conspecifics early in life (Schausberger, Gratzer, & Strodl, 2017). Adult *P. persimilis* were shown to memorize early‐life stimuli that convey information about nutritional (Rahmani et al., 2009; Zach et al., 2012), predatory (Schausberger et al., 2024) and social (Schausberger, Gratzer, & Strodl, 2017; Strodl & Schausberger, 2012, 2013) environments and adjust their behaviours accordingly. With the exception of Schausberger et al. (2024), who examined changes in the mites' personalities as a function of intraguild predation risk early in life, all studies examined mean trait expression but did not address behavioural repeatability and personality formation. Here, we assayed the mean behavioural trait expressions and repeatability of adult *P. persimilis* females reared, from hatching to pre‐adult, either in complete isolation from other conspecifics (referred to as isolated) or together with conspecifics (referred to as grouped), and mated with a male that had been either isolated or grouped since hatching.

## 2 MATERIALS AND METHODS

### 2.1 Study animals' origin and rearing

The predatory mites, *P. persimilis* (Figure 1a), used in experiments were derived from a laboratory‐reared population founded by specimens (~300) collected on eggplant, *Solanum melongena*, in Palermo, Sicily. *P. persimilis* are native in subtropical and Mediterranean climate regions in S‐America, Africa and Europe (De Moraes et al., 2004). In the laboratory, *P. persimilis* was reared in piles of detached leaves of common bean plants, *Phaseolus vulgaris*, infested by two‐spotted spider mites, *Tetranychus urticae*, that is, prey mites, on acrylic platforms. Each rearing unit was composed of a 14 × 14 × 0.2 cm platform resting on a water‐soaked foam block (14 × 14 × 5 cm) inside a plastic box (20 × 20 × 6 cm) half‐filled with tap water. Fresh spider mite‐infested leaves were added onto the platforms twice a week. To avoid contamination of the rearing by other organisms, each rearing unit was placed in a plastic tray (40 × 30 × 8 cm), containing a shallow layer of soapy water on the bottom, and covered by a translucent acrylic case with a mesh‐covered ventilation opening (10 cm Ø) on top. The laboratory population of two‐spotted spider mites, *T. urticae* (green form), was founded by specimens (~5000) collected on *P. vulgaris* in a greenhouse in Vienna. In the laboratory, the spider mites were reared on whole common bean plants, *P. vulgaris* var. Maxi, grown in a peat moss/sand mixture in 12 cm Ø pots. The predatory mite rearing units and the bean plants were kept in an air‐conditioned room at 23 ± 1°C, 50–60% RH and a 16:8 L:D photoperiod maintained by LED grow lights (SANlight FLEX 20).

### 2.2 Early‐life treatments

In the experiments, we assayed the behaviour of adult *P. persimilis* females that were either reared in isolation (referred to as ‘isolated’) or in a group (referred to as ‘grouped’) from egg hatch through the larval, protonymphal and early deutonymphal stages. All experimental animals had the same genetic background and only differed in the social treatment experienced early in life. To obtain eggs giving rise to experimental animals, gravid predatory mite females were randomly withdrawn from the rearing units and placed in groups of 15 on detached bean leaves (~15 × 10 cm) harbouring mixed spider mite stages. Detached primary leaves of common bean rested abaxial side up on moist filter paper covering a water‐soaked foam block (14 × 14 × 4 cm) inside a plastic box (20 × 20 × 6 cm) half‐filled with water. Moist tissue paper covered the petiole and the edges of the leaf to maintain turgidity and prevent escaping of the predatory mite females. Eggs laid by the predatory mite females were collected after 24 h and transferred to circular leaf discs harbouring spider mite prey. Leaf discs were punched out from trifoliate bean leaves using a cork borer and measured either 1.0 (small) or 2.3 (large) cm in diameter. Small discs harboured one adult spider mite female and her eggs laid within the preceding 24 h; large discs harboured five adult spider mite females and their eggs laid within the preceding 24 h. Different leaf disc sizes and numbers of prey were used to standardize and harmonize the leaf area and prey amount available for each predatory mite. Half of the predatory mite eggs were randomly placed in groups of six to eight on large discs (for the grouped treatment), and the other half of the eggs were placed singly on small discs (for the isolated treatment). Discs were checked once a day for the developmental progress of the predatory mites. The development of *P. persimilis* from egg to adult takes about 7 days at 23°C. Predatory mites that had reached the early deutonymphal stage were singly transferred from the disc to a closed acrylic cage, harbouring mixed spider mites as prey, and left there until they reached adulthood. Thus, one half of *P. persimilis* (grouped) were reared on leaf discs in physical presence of conspecific individuals throughout the egg, larval, protonymphal and early deutonymphal stages; the other half of *P. persimilis* (isolated) were reared from egg to early deutonymph on discs without any conspecifics. Once the predatory mite females reached adulthood inside the acrylic cage, one male mate, either isolated or grouped, was added to each cage for 24 h. After 24 h, males were discarded and each gravid female was singly transferred from the cage to a new small (1.0 cm diameter) leaf disc harbouring three adult spider mite females and their eggs laid within the preceding 24 h. This leaf disc was considered the home disc of each female, where she was kept before and in between the behavioural assays until the end of the experiment. Prey on the discs was provided in surplus and replenished as needed. Sample sizes were 150 grouped females (66 mated an isolated male, 84 mated to a grouped male) and 133 isolated females (70 mated to an isolated male, 63 mated to a grouped male).

### 2.3 Behavioural assays

To assess the repeatability of within‐group behaviours, such as movement patterns and mutual interactions including sociability, each female was subjected to four group assays. Group assays were divided into two types, referred to as mixed (groups composed of isolated and grouped females) and pure (groups composed of either isolated or grouped females). In each group assay, females were placed in groups of six into a white or greyish rectangular acrylic arena (2.8 × 1.5 cm; Figure 1b), allowed to acclimatize for 5 min, and then their behaviour was videotaped for 5 min using a digital microscope (Leica DMS1000; objective achromat 0.32, zoom setting 2.0, 24″ monitor). Each arena was delineated by moist tissue paper and harboured spider mite eggs evenly distributed across the arena (Figure 1b). In the first and third assay (both mixed type), three isolated plus three grouped females were combined in the arena, while in the second and fourth assay (both pure type), six isolated or six grouped females were combined. Before group assays, each female was marked with a tiny water colour dot on her dorsal shield. Each set of females videotaped in the group assays consisted of six differently coloured females; colours were randomly assigned; if a female lost her colour while she was on her home disc, she was re‐coloured before the next group assay. After each group assay, the females were immediately returned to their home discs. Intervals between group assays were 1–2 days. Care was taken to ensure that each female experienced a completely different set of females during each assay and that about half of the females of each set (3 ± 1) were mated to an isolated and grouped male.

To assess the repeatability of activity, each female was observed on her home disc three times within 1 h (0, 30 and 60 min) each on five single days before and in between the group assays. At each observation, the female was scored as either moving (ambulating) or being stationary; spot observations were used to calculate the proportion of time moving. Estimating the proportion of time moving by spot observations or video analysis of moving/stationary yield qualitatively and quantitatively similar results.

### 2.4 Video analysis

Each video of the group assays (set at 15 frames/s) was automatically analysed using version 3.1.0 of AnimalTA (Chiara & Kim, 2023). AnimalTA allows tracking the movement, trajectories and inter‐individual interactions separately for each individual of a group (Figure 1b,c; Data S1). The trajectories of each individual target obtained by video‐tracking (Figure 1b,c) were used for analysis of the proportion of time spent moving (moving threshold was 0.2 mm/s), area explored (individual‐centred; 2 mm^2^), moving speed (Figure 1d), meandering (an indicator of changes in moving direction), inter‐individual distance (Figure 1e), number of neighbours (neighbouring defined to be in close proximity of the target, i.e. within a radius of 2 mm from the target) (Figure 1f), which is the touching distance when both interactants longitudinally stretch their first pair of legs (Schausberger et al., 1999) and the proportion of time spent with at least 1 neighbour (Figure 1f).

### 2.5 Statistical analysis

All analyses were conducted using IBM SPSS Statistics v. 29.0.1.0 (IBM Corporation, 2023). For analyses of the population means, we used the data of all four group assays; for personality evaluations we used the data of the second, third and fourth group assay because some individuals of the first assay could not be properly tracked due to leaving the arena or technical deficiencies in detectability and/or tracking, arising from poor coloration or insufficient illumination or other technical problems.

Separate generalized estimating equations (GEEs; normal distribution) were used to analyse the effects of female early‐life treatment (isolated or grouped), male mate early‐life treatment (isolated or grouped) and type of group test (mixed or pure) on the mean proportion of time spent moving, moving speed, area explored, meandering, inter‐individual distance, number of neighbours and proportion of time with at least one neighbour. A generalized linear model (GLM; normal distribution) was used to examine the effects of the early life treatment (isolated or grouped) of the female and her mate on the total number of eggs produced in the home cages. In both GEE and GLM, we started with the full model (Data S2) and removed interaction terms with *p* ≥ 0.1 until arriving at the most parsimonious model.

**FIGURE 2:**
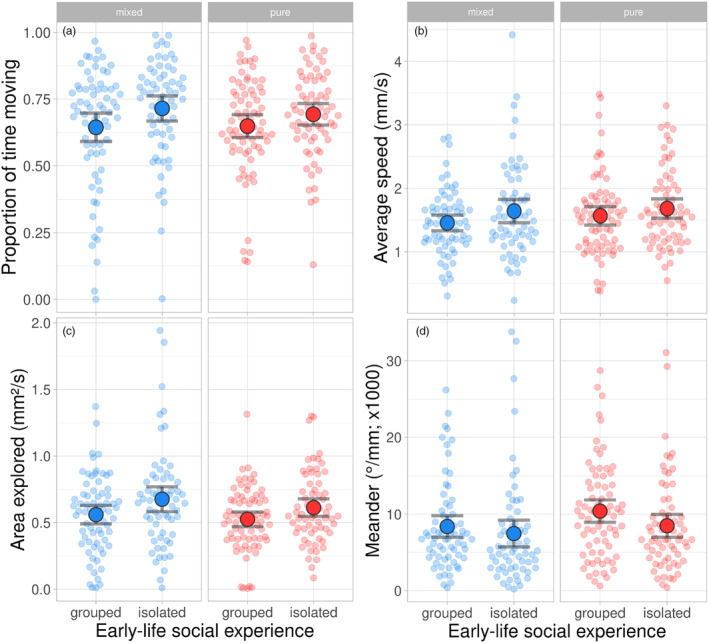
Within‐group behaviours. Mean (large circles; ±95% CI) proportion of time moving (a), moving speed (b), area explored (c) and meandering (d) of adult *Phytoseiulus persimilis* females as affected by the social experience early in life (grouped or isolated) and the type of group assay (mixed or pure). Females had been reared during early life in isolation (isolated) or in the presence of conspecifics (grouped). After reaching adulthood and mating, each female was subjected to four group assays (two of each type), where six females were combined in an acrylic arena. Mixed refers to sets consisting of three females that had been isolated and three females that had been grouped early in life; pure refers to sets consisting of either six females that had been isolated or six females that had been grouped early in life. Data were extracted from videos using AnimalTA (Chiara & Kim, 2023). Small semi‐transparent dots are the individual data; statistical analysis is in Table 1.

**FIGURE 3:**
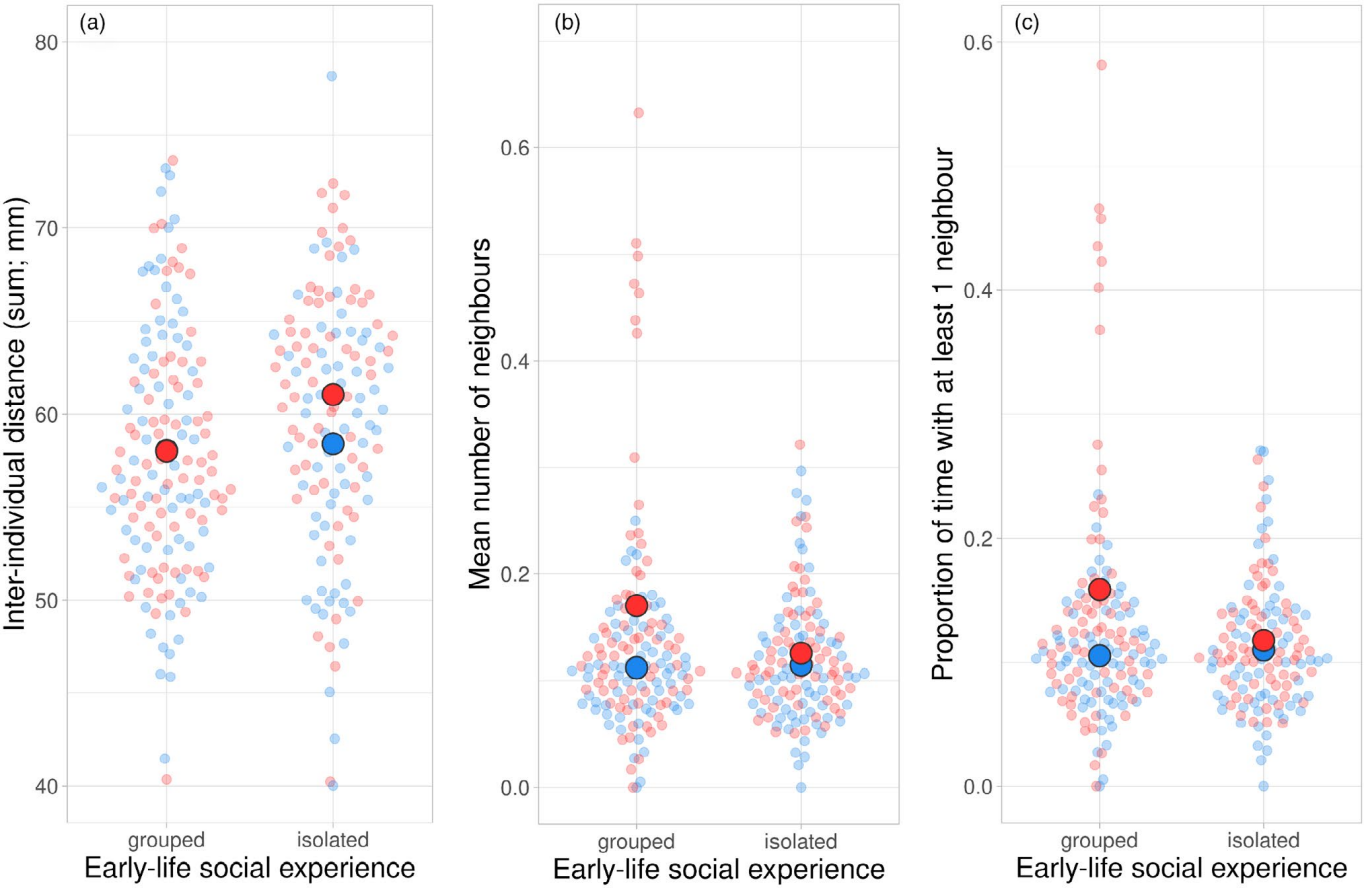
Sociability traits. Mean (large circles) inter‐individual distance (a), number of neighbours (b) and proportion of time with at least one neighbour (c) of adult *Phytoseiulus persimilis* females as affected by the social experience made early in life (grouped or isolated) and the type of group assay (mixed in blue; pure in red). Females had been reared during early life in isolation (isolated) or in the presence of conspecifics (grouped). After reaching adulthood and mating, each female was subjected to four group assays (two types), where six females were combined in an acrylic arena. Mixed refers to sets consisting of three females that had been isolated and three females that had been grouped early in life; pure refers to sets consisting of either six females that had been isolated or six females that had been grouped early in life. Data were extracted from videos using AnimalTA (Chiara & Kim, 2023). Small semi‐transparent dots are the individual data; statistical analysis is in Table 1.

Personality expression, that is, behavioural consistency across group assays, in the proportion of time spent moving, moving speed, area explored, meandering, area explored, inter‐individual distance, number of neighbours and proportion of time with at least one neighbour was evaluated by intraclass correlation coefficients (ICC), indicating behavioural repeatability (two‐way random, consistency, average measure; McGraw & Wong, 1996). ICCs were calculated for all females pooled and females split into isolated and grouped and females mated to isolated and grouped males. All females pooled represents a situation where some individuals experience social contact early in life and others not. Within‐ and among‐individual variances were checked to pinpoint the causes of different ICCs induced by early social experience of the female (isolated vs. grouped) and/or their mates (isolated vs. grouped). SuperPlotsOfData (Goedhart, 2021) was used to create Figures 2 and 3.

## 3 RESULTS

### 3.1 Population means

The social environment early in life had a significant effect on each behavioural trait extracted from video‐tracking (Table 1; Figures 2 and 3). Grouped females were less active, moved at slower speeds, explored a smaller area, meandered more and kept shorter distances to other group members than isolated females (Figures 2a–d and 3a). In pure but not mixed groups, grouped females had more close neighbours (within a 2‐mm radius) and spent more time with close neighbours than isolated females (Table 1; Figure 3b,c). The early social environment experienced by male mates did not influence the mean behavioural traits of the females. Mean moving activity of the females in their home cages did not vary with the social environment of either the female or her mate (GLM; Wald *χ* ^2^ = 0.040, *p* = 0.842 for females and Wald *χ* ^2^ = 0.458, *p* = 0.498 for male mates). Egg production in the home cages (mean ± SE; females isolated 22.49 ± 0.92, grouped 22.61 ± 0.87; males isolated 22.39 ± 0.91, grouped 22.72 ± 0.89) was neither influenced by the early social environment of the females nor that of their mates (GLM; Wald *χ* ^2^ = 0.009, *p* = 0.926 for females and Wald *χ* ^2^ = 0.067, *p* = 0.796 for male mates).

**TABLE 1:** Results of generalized estimating equations (GEE; most parsimonious models) of the effects of the early social treatments of females and their mates (grouped or isolated) and the type of group assay (mixed or pure) on mean trait expressions of adult predatory mite females *Phytoseiulus persimilis*.

Dependent variable	Independent variables	*N*	Wald *χ* ^2^	df	*p*‐Value^a^
Inter‐individual distance	(Intercept)	274	18089.575	1	<0.001
	Female treatment		**3.810**	**1**	**0.050**
	Male treatment		0.033	1	0.855
	Type of test		2.352	1	0.125
Number of neighbours	(Intercept)	274	599.274	1	<0.001
	Female treatment		**3.959**	1	**0.047**
	Male treatment		0.326	1	0.568
	Type of test		**14.251**	**1**	**<0.001**
	Female treatment × type of test		**6.683**	**1**	**0.010**
Time with ≥1 neighbour	(Intercept)	274	718.066	1	<0.001
	Female treatment		**4.066**	1	**0.044**
	Male treatment		0.236	1	0.627
	Type of test		**13.625**	**1**	**<0.001**
	Female treatment × type of test		**7.636**	**1**	**0.006**
Time moving	(Intercept)	270	3595.649	1	<0.001
	Female treatment		**6.882**	**1**	**0.009**
	Male treatment		1.304	1	0.253
	Type of test		0.143	1	0.706
Average speed	(Intercept)	273	1773.503	1	<0.001
	Female treatment		**4.239**	**1**	**0.039**
	Male treatment		1.008	1	0.315
	Type of test		1.063	1	0.303
Meander	(Intercept)	271	525.864	1	<0.001
	Female treatment		**3.831**	**1**	**0.050**
	Male treatment		0.471	1	0.493
	Type of test		**4.316**	**1**	**0.038**
	Male treatment × type of test		3.143	1	0.076
Area explored	(Intercept)	274	1143.976	1	<0.001
	Female treatment		**9.135**	**1**	**0.003**
	Male treatment		2.804	1	0.094
	Type of test		1.919	1	0.120

^a^ Significant effects of independent variables (*p* ≤ 0.05) are highlighted in bold.

### 3.2 Intraclass correlation coefficients

Grouped females showed personalities in five of seven behavioural traits measured in the group assays, while the behaviour of isolated females was only repeatable in the number of neighbours (Table 2). Depending on the trait, higher repeatability in grouped females was caused by higher among‐individual variability, lower within‐individual variability or both. The ICCs of both grouped and isolated females pooled were repeatable in two of seven traits. While activity measured in the group assays was only repeatable in grouped but not isolated females (Table 2), activity measured in the home cages, where the predatory mite females were kept singly, was only repeatable in isolated (ICC = 0.571, 95% CI 0.269–0.773, *p* < 0.001) but not grouped (ICC = −0.107, 95% CI −0.815 to 0.382, *p* = 0.623) females. Also, the social environment experienced by the male mates of the females influenced female personality expression: four of seven traits measured in the group assays were repeatable in females mated to isolated males but none were repeatable in females mated to grouped males (Table 3). This was exclusively due to lower within‐individual variability in females mated to isolated males. Moving activity as measured in the home cages, where each female was alone, was highly repeatable in females mated to isolated males (ICC = 0.483, 95% CI 0.094–0.713, *p* = 0.006) but was not repeatable in those mated to grouped males (ICC = 0.085, 95% CI −0.376 to 0.539, *p* = 0.352).

**TABLE 2:** Intraclass correlation coefficients (ICC; two‐way random, consistency; average measure) of behavioural traits of experimental females as affected by the early social treatment (grouped or isolated).

Personality trait	Early life treatment of females	*N*	ICC^a^	CI (95%)^a^	*p*‐Value^a^
Inter‐individual distance	Grouped	35	**0.378**	**−0.091**; **0.665**	**0.049**
	Isolated	31	−0.028	−0.866; 0.470	0.520
	Pooled	66	0.192	−0.217; 0.479	0.153
Number of neighbours	Grouped	35	−0.398	−1.450; 0.247	0.857
	Isolated	31	**0.457**	**0.014**; **0.720**	**0.022**
	Pooled	66	−0.199	−0.804; 0.227	0.790
Time with ≥1 neighbour	Grouped	35	−0.430	−1.507; 0.230	0.873
	Isolated	31	0.373	−0.138; 0.677	0.062
	Pooled	66	−0.256	−0.890; 0.191	0.846
Time moving	Grouped	35	**0.403**	**−0.045**; **0.679**	**0.036**
	Isolated	31	0.017	−0.784; 0.493	0.464
	Pooled	66	0.283	−0.080; 0.538	0.056
Average speed	Grouped	35	**0.688**	**0.453**; **0.832**	**<0.001**
	Isolated	31	0.330	−0.216; 0.655	0.094
	Pooled	66	**0.516**	**0.272**; **0.688**	**<0.001**
Meander	Grouped	34	**0.461**	**0.048**; **0.713**	**0.017**
	Isolated	31	0.062	−0.703; 0.516	0.407
	Pooled	65	**0.302**	**−0.054**; **0.552**	**0.044**
Area explored	Grouped	35	**0.421**	**−0.015**; **0.688**	**0.028**
	Isolated	31	0.037	−0.749; 0.503	0.439
	Pooled	66	0.250	−0.129; 0.517	0.084

^a^ Significant ICCs (*p* ≤ 0.05) are highlighted in bold.

**TABLE 3:** Intraclass correlation coefficients (ICC; two‐way‐random, consistency; average measure) of behavioural traits of *Phytoseiulus persimilis* females as affected by the early social treatment of their male mates (grouped or isolated).

Personality trait	Early social treatment of male mates	*N*	ICC^a^	CI (95%)^a^	*p*‐Value^a^
Inter‐individual distance	Grouped	34	0.257	0.313; 0.604	0.152
	Isolated	32	0.025	−0.753; 0.492	0.454
Number of neighbours	Grouped	34	−0.278	−1.258; 0.319	0.778
	Isolated	32	−0.006	−0.808; 0.476	0.493
Time with ≥1 neighbour	Grouped	34	−0.298	−1.293; 0.308	0.792
	Isolated	32	−0.140	−1.050; 0.405	0.648
Time moving	Grouped	34	0.100	−0.590; 0.521	0.351
	Isolated	32	**0.532**	**0.158**; **0.786**	**0.006**
Average speed	Grouped	34	0.370	−0.113; 0.664	0.056
	Isolated	32	**0.662**	**0.392**; **0.824**	**<0.001**
Meander	Grouped	34	0.127	−0.542; 0.535	0.313
	Isolated	31	**0.593**	**0.261**; **0.790**	**0.002**
Area explored	Grouped	34	0.112	−0.582; 0.532	0.336
	Isolated	32	**0.423**	**−0.029**; **0.695**	**0.031**

^a^ Significant ICCs (*p* ≤ 0.05) are highlighted in bold.

## 4 DISCUSSION

Our study documents that early social isolation has persistent, profound influences on mean behavioural traits and personality expression of adult predatory mite females *P. persimilis*. Both mean trait expression and personality expression suggest that early social isolation has disruptive effects on the way the mites behave within groups. *P. persimilis* are adapted to living in groups, with experience of social interactions being the typical state and social isolation being a highly stressful condition (Schausberger, 2004, 2005; Schausberger, Gratzer, & Strodl, 2017). On average, socially isolated females were more restless, ran more quickly, explored a larger area, meandered less, had fewer close neighbours, spent less time with close neighbours and kept larger inter‐individual distances than socially experienced females. When assayed in groups, adult females showed repeatability in activity, moving speed, meandering, area explored and inter‐individual distance. In contrast, when the mites had been deprived of any social contact early in life, adult personality expression was disrupted in the presence of conspecifics, and the above‐listed traits were no longer repeatable. When assayed in isolation, females that had been socially isolated early in life were consistent in activity whereas females that had experienced social interactions early in life were not. Strikingly, the early social environment of the females' mates had strong effects on female personalities, but not on mean trait expression. Mating with socially isolated males strongly increased the behavioural repeatability of socially experienced females.

The effects of early social isolation on mean trait expressions in behaviour, physiology and/or life history later in life are documented for numerous animal species (e.g. Cacioppo et al., 2015; Sachser et al., 2011; Schausberger, 2004; Snyder‐Mackler et al., 2020; Vora et al., 2022). Alterations in traits expressed in groups, such as greater mean restlessness, less meandering and faster moving speed, linked to larger areas explored, are not necessarily detrimental in themselves, but indicate that the presence of conspecifics represents a stressful situation for isolation‐reared mites, affecting their social homeostasis (Matthews & Tye, 2019). Increased locomotion time and speed are often interpreted as hyperactivity such as observed in socially deprived rats (Levine et al., 2007) and angelfish (Gomez‐Laplaza & Morgan, 1991). Analogous to larger inter‐individual distances and fewer close neighbours in isolated than grouped predatory mite females (present study; Schausberger, Gratzer, & Strodl, 2017), Shams et al. (2017) observed larger inter‐individual distances and Groneberg et al. (2020) observed stronger avoidance reactions to conspecifics in socially isolated zebrafish larvae. Altogether, the observed changes in mean trait expressions of socially deprived individuals relative to socially experienced individuals support the interpretation that early social isolation disrupts the maintenance of personality expression in groups. Similarly, social isolation disrupts typical social behaviour and interferes with brain development and gene expression in bumble bees (Wang et al., 2022).

Recent studies emphasized that the expression of personality may vary among social contexts under which a given personality trait is measured (Belgrade & Griffen, 2017 for crabs; Brand et al., 2022 for lizards; Planas‐Sitja & Deneubourg, 2018 for cockroaches). Our study suggests that the early social experience and the match between the early experience and the social conditions during testing may determine whether personality traits, such as activity, are repeatable. Group‐experienced individuals were highly repeatable in activity (proportion of time moving quantified by video analysis) when tested in a group but not when tested alone, whereas the opposite was true for isolation‐experienced individuals, which were highly repeatable in activity (proportion of time moving quantified by repeated spot observations) when tested alone but not when tested in a group. Similarly, Jolles et al. (2016) showed that for sticklebacks, social housing conditions experienced just prior to personality assays can critically influence the repeatability of personality traits such as boldness, with isolation‐experienced individuals being more repeatable than group‐experienced individuals when tested in isolation.

Early‐life social experience increasing repeatability supports the idea that social interactions within groups are major drivers of individualized social niche formation (Bergmüller & Taborsky, 2010; Mutwill et al., 2023). However, the fact that isolated but not grouped females were highly repeatable in activity when tested in isolation counters this explanation or at least begs for an alternative explanation. Matching social environments during early life and adult personality assays is a plausible explanation. Along the same line, several studies suggest that experiencing altered social environments promotes behavioural plasticity, whereas experiencing consistent social environments promotes behavioural consistency (Riley et al., 2018 for skinks; Munson et al., 2021 for sticklebacks; Skinner & Miller, 2022 for snakes).

Personalities may change with important life‐history events, such as developmental transitions, maturation and onset of reproduction (e.g. Wilson & Krause, 2012); among life‐history events, the experience of mating, including the phenotype of the first mate, has critical relevance (e.g. Monestier & Bell, 2020). This is especially true for animals that have the potential to mate multiply, which results in indirect but no direct benefits, such as in *P. persimilis* (Schausberger et al., 2016; Schausberger, Walzer, et al., 2017); from the first mate experience, females can decide whether another mate would likely be available or not, and whether multiple mating would be favourable or not. This information may decisively influence behavioural consistency and personality expression of the females. Monestier and Bell (2020) compared the effects of experiencing mating versus courtship only versus no mating on female personality. The effects of the mate phenotype on female personality expression have not yet been examined. Male *P. persimilis* differ in mating behaviour when having been grouped or socially isolated early in life, with isolated males initiating mating more quickly (Schausberger, Walzer, et al., 2017). In our experiments, male early‐life experience severely affected the personalities of their female mates but did not have any effect on mean trait expressions. Mate‐induced changes in female personality expression may have been due to male manipulation, female response to the male or both (e.g. Bath et al., 2021). Isolated males induced very high repeatability in grouped but not isolated females, which rather points at the interaction between female and male phenotype or exclusive female response to the male phenotype than at exclusive male manipulation. Females could have interpreted perception of the isolated state of their mate as information that alternative or additional mates are lacking or rare. Accordingly, having no expectation to mate again, such females become more consistent in their behaviour; it does not pay for them to be more plastic, they had a mate and the chance that other mates are around is low (as perceived from the information conveyed by the isolated mate). *P. persimilis* females gain indirect benefits from multiple mating (Schausberger et al., 2016, Schausberger, Walzer, et al., 2017) and multiply mating females typically go for males that are different from the first mate (Enigl & Schausberger, 2004); such behaviour requires plastic mate choice and acceptance (Atalay & Schausberger, 2018; Schausberger & Çekin, 2020), which may be coupled to plasticity in other behavioural traits (e.g. Nielsen & Papaj, 2022; Sheehy & Laskowski, 2023), as seen in females mated to socially experienced males. Alternatively, changes in female personality expression could have been caused by male manipulation to enhance their reproductive success by making the females to go less likely for another male, but socially isolated females were less manipulatable owing to their own early isolation experience overshadowing manipulation by the male mate. In contrast, grouped males convey the information that other males are nearby, and in such a case, it pays for the females to expect additional mating opportunities and to be more flexible in mate choice and acceptance, as well as other within‐group behaviours. Possible proximate mechanisms by which females recognized the mates' state (socially isolated early in life or not) include chemical signatures in seminal fluids (Perry et al., 2013), body odours (Dicke, 2014) and/or behavioural traits (Munson et al., 2020). Polyandrous females typically become more selective with mating experience (Jennions & Petrie, 1997). Life‐history theories concerned with the adaptive evolution of personality traits predict that the mating experience influences a female's willingness to take risks and to become either more plastic or more consistent, depending on the information conveyed by the mate and her residual reproductive value (Wolf et al., 2007).

Potential benefits arising from expressing and maintaining personalities within groups include enhanced group functioning and social network dynamics (e.g. Gartland et al., 2022; Jeanson & Weidenmüller, 2014; Jolles et al., 2018). Most importantly, having different personality types within groups reduces inter‐individual conflicts, owing to different personality types occupying different individualized social niches within groups. These effects should also manifest at higher organizational levels, such as populations and communities, but this remains to be evaluated.

## AUTHOR CONTRIBUTIONS

Peter Schausberger conceived the study idea, acquired funding and supervised the research activities; Peter Schausberger and Thi Hanh Nguyen designed the experiments and analysed the data; Thi Hanh Nguyen conducted the experiments and took care of the rearing; Peter Schausberger wrote the first draft of the manuscript; Peter Schausberger and Thi Hanh Nguyen contributed to revision, read the final version of the manuscript and approved its submission.

## CONFLICT OF INTEREST STATEMENT

The authors declare no conflict of interest.

## STATEMENT ON INCLUSION

Our study brings together authors from different countries, including scientists based in the country where the study was carried out. All authors were engaged early on with the research and study design to ensure that the diverse sets of perspectives they represent were considered from the onset.

## Supporting information


**Data S1.** Demonstration video of mite tracking.

**Data S2.** Full models of generalized estimating equations (GEEs).

## Data Availability

Data available from figshare https://figshare.com/s/10750d4412dcd6cde0d7 (Schausberger & Nguyen, 2024).
