# Supplementary material for: Early social isolation disrupts adult personality expression in group‐living mites
Source: J Anim Ecol. 2024 Aug 23;94(1):45–57. doi: 10.1111/1365-2656.14169 (PMC11730261; doi:10.1111/1365-2656.14169)
Supplement: Supplementary file 2 — Data S2. Full models of generalized estimating equations (GEEs). [file JANE-94-45-s002.pdf]

Generalized Estimating Equations: inter-individual distances

Categorical Variable Information

			N	Percent
Factor	FemTreatment	isolated	130	47.4%
		grouped	144	52.6%
		Total	274	100.0%
	MaleTreatment	isolated	134	48.9%
		grouped	140	51.1%
		Total	274	100.0%
	Test_Type	mixed	134	48.9%
		pure	140	51.1%
		Total	274	100.0%

Tests of Model Effects

	Type III		
Source	Wald Chi-Square	df	Sig.
(Intercept)	17937.216	1	<.001
FemTreatment	3.634	1	.057
MaleTreatment	.022	1	.883
Test_Type	2.908	1	.088
FemTreatment * MaleTreatment	.304	1	.581
FemTreatment * Test_Type	2.971	1	.085
MaleTreatment * Test_Type	.014	1	.905
FemTreatment * MaleTreatment * Test_Type	.257	1	.612

Dependent Variable: Mean_sum_interind_distances_mean
Model: (Intercept), FemTreatment, MaleTreatment, Test_Type, FemTreatment * MaleTreatment, FemTreatment * Test_Type, MaleTreatment * Test_Type, FemTreatment * MaleTreatment * Test_Type

Generalized Estimating Equations: mean number of neighbors

Categorical Variable Information

			N	Percent
Factor	FemTreatment	isolated	130	47.4%
		grouped	144	52.6%
		Total	274	100.0%
	MaleTreatment	isolated	134	48.9%
		grouped	140	51.1%
		Total	274	100.0%
	Test_Type	mixed	134	48.9%
		pure	140	51.1%
		Total	274	100.0%

Tests of Model Effects

Source	Type III Wald Chi-Square	df	Sig.
(Intercept)	647.353	1	<.001
FemTreatment	3.997	1	.046
MaleTreatment	.337	1	.561
Test_Type	14.650	1	<.001
FemTreatment * MaleTreatment	.001	1	.981
FemTreatment * Test_Type	6.741	1	.009
MaleTreatment * Test_Type	.346	1	.556
FemTreatment * MaleTreatment * Test_Type	.171	1	.679

Dependent Variable: Mean_nb_neighbours_mean
Model: (Intercept), FemTreatment, MaleTreatment, Test_Type, FemTreatment * MaleTreatment, FemTreatment * Test_Type, MaleTreatment * Test_Type, FemTreatment * MaleTreatment * Test_Type

Generalized Estimating Equations. proportion of time with at least 1 neighbor

Categorical Variable Information

			N	Percent
Factor	FemTreatment	isolated	130	47.4%
		grouped	144	52.6%
		Total	274	100.0%
	MaleTreatment	isolated	134	48.9%
		grouped	140	51.1%
		Total	274	100.0%
	Test_Type	mixed	134	48.9%
		pure	140	51.1%
		Total	274	100.0%

Tests of Model Effects

Source	Type III Wald Chi-Square	df	Sig.
(Intercept)	784.414	1	<.001
FemTreatment	4.108	1	.043
MaleTreatment	.241	1	.623
Test_Type	14.091	1	<.001
FemTreatment * MaleTreatment	.000	1	1.000
FemTreatment * Test_Type	7.729	1	.005
MaleTreatment * Test_Type	.364	1	.546
FemTreatment * MaleTreatment * Test_Type	.057	1	.812

Dependent Variable: Prop_time_with_at_least_one_neighbour_mean
Model: (Intercept), FemTreatment, MaleTreatment, Test_Type, FemTreatment * MaleTreatment, FemTreatment * Test_Type, MaleTreatment * Test_Type, FemTreatment * MaleTreatment * Test_Type

Generalized Estimating Equations: proportion of time moving

Categorical Variable Information

			N	Percent
Factor	FemTreatment	isolated	130	47.4%
		grouped	144	52.6%
		Total	274	100.0%
	MaleTreatment	isolated	134	48.9%
		grouped	140	51.1%
		Total	274	100.0%
	Test_Type	mixed	134	48.9%
		pure	140	51.1%
		Total	274	100.0%

Tests of Model Effects

Source	Type III		
	Wald Chi-Square	df	Sig.
(Intercept)	3618.087	1	<.001
FemTreatment	6.936	1	.008
MaleTreatment	1.452	1	.228
Test_Type	.128	1	.721
FemTreatment * MaleTreatment	1.426	1	.232
FemTreatment * Test_Type	.258	1	.611
MaleTreatment * Test_Type	2.548	1	.110
FemTreatment * MaleTreatment * Test_Type	1.059	1	.303

Dependent Variable: Prop_time_moving_mean
Model: (Intercept), FemTreatment, MaleTreatment, Test_Type, FemTreatment * MaleTreatment, FemTreatment * Test_Type, MaleTreatment * Test_Type, FemTreatment * MaleTreatment * Test_Type

Generalized Estimating Equations : moving speed

Categorical Variable Information

			N	Percent
Factor	FemTreatment	isolated	130	47.6%
		grouped	143	52.4%
		Total	273	100.0%
	MaleTreatment	isolated	133	48.7%
		grouped	140	51.3%
		Total	273	100.0%
	Test_Type	mixed	133	48.7%
		pure	140	51.3%
		Total	273	100.0%

Tests of Model Effects

Source	Type III Wald Chi-Square	df	Sig.
(Intercept)	1145.076	1	<.001
FemTreatment	2.724	1	.099
MaleTreatment	.806	1	.369
Test_Type	1.134	1	.287
FemTreatment * MaleTreatment	.803	1	.370
FemTreatment * Test_Type	.271	1	.603
MaleTreatment * Test_Type	.031	1	.861
FemTreatment * MaleTreatment * Test_Type	.018	1	.893

Dependent Variable: Average_Speed_Moving_mean
Model: (Intercept), FemTreatment, MaleTreatment, Test_Type, FemTreatment * MaleTreatment, FemTreatment * Test_Type, MaleTreatment * Test_Type, FemTreatment * MaleTreatment * Test_Type

Generalized Estimating Equations : meandering

Categorical Variable Information

			N	Percent
Factor	FemTreatment	isolated	130	48.0%
		grouped	141	52.0%
		Total	271	100.0%
	MaleTreatment	isolated	132	48.7%
		grouped	139	51.3%
		Total	271	100.0%
	Test_Type	mixed	132	48.7%
		pure	139	51.3%
		Total	271	100.0%

Tests of Model Effects

	Type III		
Source	Wald Chi-Square	df	Sig.
(Intercept)	436.058	1	<.001
FemTreatment	3.089	1	.079
MaleTreatment	.439	1	.507
Test_Type	4.082	1	.043
FemTreatment * MaleTreatment	.263	1	.608
FemTreatment * Test_Type	.671	1	.413
MaleTreatment * Test_Type	3.769	1	.052
FemTreatment * MaleTreatment * Test_Type	1.046	1	.306

Dependent Variable: Meander_mean
Model: (Intercept), FemTreatment, MaleTreatment, Test_Type, FemTreatment * MaleTreatment, FemTreatment * Test_Type, MaleTreatment * Test_Type, FemTreatment * MaleTreatment * Test_Type

Generalized Estinating Equations : area explored

Categorical Variable Information

			N	Percent
Factor	FemTreatment	isolated	130	47.4%
		grouped	144	52.6%
		Total	274	100.0%
	MaleTreatment	isolated	134	48.9%
		grouped	140	51.1%
		Total	274	100.0%
	Test_Type	mixed	134	48.9%
		pure	140	51.1%
		Total	274	100.0%

Tests of Model Effects

	Type III		
Source	Wald Chi-Square	df	Sig.
(Intercept)	1094.973	1	<.001
FemTreatment	8.819	1	.003
MaleTreatment	2.904	1	.088
Test_Type	1.765	1	.184
FemTreatment * MaleTreatment	1.100	1	.294
FemTreatment * Test_Type	.150	1	.699
MaleTreatment * Test_Type	.148	1	.701
FemTreatment * MaleTreatment * Test_Type	1.290	1	.256

Dependent Variable: ExploAbs_PerSec
Model: (Intercept), FemTreatment, MaleTreatment, Test_Type, FemTreatment * MaleTreatment, FemTreatment * Test_Type, MaleTreatment * Test_Type, FemTreatment * MaleTreatment * Test_Type
